# Detachment of the RNA degradosome from the inner membrane of *Escherichia coli* results in a global slowdown of mRNA degradation, proteolysis of RNase E and increased turnover of ribosome‐free transcripts

**DOI:** 10.1111/mmi.14248

**Published:** 2019-04-06

**Authors:** Lydia Hadjeras, Leonora Poljak, Marie Bouvier, Quentin Morin‐Ogier, Isabelle Canal, Muriel Cocaign‐Bousquet, Laurence Girbal, Agamemnon J. Carpousis

**Affiliations:** ^1^ Laboratoire de Microbiologie et de Génétique Moléculaires, Centre de Biologie Intégrative (CBI) Université de Toulouse, CNRS, UPS Toulouse France; ^2^ LISBP Université de Toulouse, CNRS, INRA, INSA Toulouse France

## Abstract

The reason for RNase E attachment to the inner membrane is largely unknown. To understand the cell biology of RNA degradation, we have characterized a strain expressing RNase E lacking the membrane attachment site (cytoplasmic RNase E). Genome‐wide data show a global slowdown in mRNA degradation. There is no correlation between mRNA stabilization and the function or cellular location of encoded proteins. The activity of cRNase E is comparable to the wild‐type enzyme *in vitro*, but the mutant protein is unstable *in vivo*. Autoregulation of cRNase E synthesis compensates for protein instability. cRNase E associates with other proteins to assemble a cytoplasmic RNA degradosome. CsrB/C sRNAs, whose stability is regulated by membrane‐associated CsrD, are stabilized. Membrane attachment of RNase E is thus necessary for CsrB/C turnover. In contrast to mRNA stability, ribosome‐free transcripts are sensitive to inactivation by cRNase E. Our results show that effects on RNA degradation are not due to the differences in the activity or level of cRNase E, or failure to assemble the RNA degradosome. We propose that membrane attachment is necessary for RNase E stability, functional interactions with membrane‐associated regulatory factors and protection of ribosome‐free transcripts from premature interactions with RNase E in the nucleoid.

## Graphical abstract

Attachment of RNase E to the inner membrane of *E. coli* is important for multiple processes including efficient degradation of mRNA, interactions with membrane‐associated regulatory factors involved in the posttranscriptional control of gene expression, and the protection of nascent transcripts in the nucleoid from degradation by RNase E. Detachment of RNase E from the inner membrane results in its proteolysis, which could be important for downregulating RNase E activity under stress conditions.

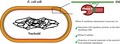

## Introduction

RNase E is an essential endo‐ribonuclease with a major role in RNA processing and degradation. A recent genome‐wide study has mapped over 22,000 RNase E cleavage sites (Chao *et al.*, 2017). In agreement with earlier work (Carpousis *et al.*, 2009; Gorna *et al.*, 2012; Mohanty and Kushner, 2016), RNase E prefers single‐stranded substrates and sequence specificity is limited to a conserved U at +2 and preference for G at −2 relative to the cleavage site. Since nearly every transcript in the cell has multiple RNase E cleavage sites, an important issue is how access to these sites is regulated during the lifetime of a transcript? RNase E has a major role in the initiation of mRNA degradation. The turnover of mRNA with half‐lives in the range of 2–3 min is an important feature of gene expression permitting rapid re‐modelling of the transcriptome in response to changes in environmental conditions (Esquerre *et al.*, 2014).

RNase E also initiates the degradation of regulatory RNA such as CsrB and CsrC, which have mRNA‐like half‐lives (Suzuki *et al.*, 2006). Carbon storage regulation (Csr) involves the mRNA binding protein CsrA, which affects translation and mRNA stability of a large regulon of RNA targets (Vakulskas *et al.*, 2015). Membrane‐associated CsrD regulates RNase E‐dependent degradation of CsrB/C. These regulatory RNAs are ‘sponges’ that bind multiple copies of CsrA. The dynamic instability of CsrB/C is an important property that can rapidly increase the level of free CsrA in the absence of protein synthesis by downregulating CsrB/C levels.

RNase E is a large 1061 residues protein composed of an N‐terminal catalytic domain and a C‐terminal region that is mostly natively unstructured (Callaghan *et al.*, 2004; Callaghan *et al.*, 2005). Embedded in the noncatalytic region are SLiMs (short linear motifs), also known as microdomains, that have the potential to form sites of interaction with other molecules. The association of RNase E with the exo‐ribonuclease, PNPase, the DEAD‐box RNA helicase, RhlB, and the glycolytic enzyme, enolase forms a multienzyme complex known as the RNA degradosome (Carpousis *et al.*, 1994; Py *et al.*, 1996; Vanzo *et al.*, 1998; Carpousis *et al.*, 1999; Carpousis, 2007; Mackie, 2013; Hui *et al.*, 2014). In this complex, RhlB interacts with RNase E and PNPase to facilitate the degradation of structured RNA.

The MTS (membrane targeting sequence) is a 15‐residue SLiM that forms an amphipathic α‐helix upon association with phospholipid bilayers. *In vitro*, the MTS makes an avid interaction with protein‐free phospholipid bilayers; *in vivo*, the MTS is necessary for attachment of RNase E to the inner cytoplasmic membrane (Khemici *et al.*, 2008; Strahl *et al.*, 2015). RNase E orthologues are ubiquitous and highly conserved in the γ‐Proteobacteria (Ait‐Bara and Carpousis, 2015; Ait‐Bara *et al.*, 2015). A large noncatalytic region with a conserved MTS located about 40 residues downstream of the catalytic domain are hallmarks of the RNase E orthologues. The conservation of the MTS strongly suggests that attachment of RNase E to the inner cytoplasmic membrane has important functional consequences for RNase E activity and specificity.

The level of RNase E in the cell is regulated posttranscriptionally (Hui *et al.*, 2014). The *rne* mRNA has a large 361 nt 5′ UTR (untranslated region) that is necessary for autoregulation of RNase E synthesis (Jain and Belasco, 1995; Diwa *et al.*, 2000; Schuck *et al.*, 2009). RNase E binds to the 5′ UTR to cleave the *rne* mRNA and thereby downregulates its synthesis. Autoregulation assures the maintenance of adequate levels of RNase E in the cell. A consequence of autoregulation is that mutations leading to decreased RNase E activity are compensated by increased synthesis resulting in higher levels of the mutant enzyme. For example, although the noncatalytic region of RNase E is dispensable, characterization of a series of mutants resulting in deletions in the noncatalytic region showed a 7.5‐fold difference in protein levels (Leroy *et al.*, 2002). This work is an example of how autoregulation compensates for mutations that affect RNase E activity.

Here, we characterize effects of the *rne*∆*MTS* mutation on RNA degradation. This allele expresses cytoplasmic RNase E (cRNase E), a mutant protein in which the membrane attachment site has been deleted (Khemici *et al.*, 2008; Strahl *et al.*, 2015). Strains with a single copy of the *rne*∆*MTS* allele in the chromosome are viable although they grow more slowly than wild type. cRNase E appears to be uniformly distributed in the interior of the cell as evidenced by epifluorescence and super‐resolution microscopy (Strahl *et al.*, 2015, Moffitt *et al.*, 2016). The rationale of this work is that characterizing defects in RNA degradation in the* rne*∆*MTS* strain should shed light on the functional consequences of the attachment of RNase E to the inner cytoplasmic membrane.

## Results

### Slow growth phenotype

We have previously shown that a plasmid copy of the *rne∆MTS* allele expressing cRNase E complements a strain with the disruption of the *rne* gene in the chromosome (Khemici *et al.*, 2008). Here, we are using a single‐copy chromosomal construct of the *rne∆MTS* allele at the *rne* locus. We transduced the *rne∆MTS* allele into two different *E. coli* backgrounds (Table S1). The main difference between MG1655 and NCM3416 is a mutation in the *rph‐pyrE* operon in MG1655 that inactivates RNase PH and knocks down *pyrE* expression levels (Jensen, 1993; Soupene *et al.*, 2003). We measured the growth of these strains under different conditions (Table 1). In general, growth in the MG1655 background is slower than in the NCM3416 background, which is likely the result of limitation of pyrimidine synthesis in MG1655 due to lower levels of *pyrE* expression (Soupene *et al.*, 2003). The strains with the* rne∆MTS* allele grow 10 to 25% more slowly than the isogenic wild‐type control under all conditions tested. The relative difference in growth rate in LB at 37°C is comparable to our previous measurements with a plasmid copy of *rne∆MTS* (Khemici *et al.*, 2008) showing that the growth defect is not an artefact of complementation with a plasmid. Slower growth of the* rne∆MTS* strain under the conditions tested shows that temperature, nutrients and strain background do not significantly affect the growth phenotype, which is therefore an intrinsic property of the *rne∆MTS* allele.

**Table 1:** Growth rates.

Strain	Background	Genotype	Medium	°C^a^	dt (min)	µ (h^−1^)	µ/µ^wt^
MG1655	MG1655	*rne*	M9‐glucose	37	64	0.65	1.00
Kti658	MG1655	*rne∆MTS frt* b	M9‐glucose	37	95	0.44	0.68
MBS106	NCM3416	*rne frt*	M9‐glucose	37	56	0.74	1.00
MBS157	NCM3416	*rne∆MTS frt*	M9‐glucose	37	63	0.66	0.89
MG1655	MG1655	*rne*	M9‐glucose‐CAA^c^	37	40	1.04	1.00
Kti658	MG1655	*rne∆MTS frt*	M9‐glucose‐CAA	37	52	0.80	0.77
MBS106	NCM3416	*rne frt*	M9‐glucose‐CAA	37	34	1.22	1.00
MBS157	NCM3416	*rne∆MTS frt*	M9‐glucose‐CAA	37	43	0.97	0.80
MG1655	MG1655	*rne*	LB	37	31	1.34	1.00
Kti658	MG1655	*rne∆MTS frt*	LB	37	42	0.99	0.74
MBS106	NCM3416	*rne frt*	LB	37	25	1.66	1.00
MBS157	NCM3416	*rne∆MTS frt*	LB	37	31	1.34	0.81
MBS106	NCM3416	*rne frt*	MOPS–glycerol–CAA	30	55	0.76	1.00
MBS157	NCM3416	*rne∆MTS frt*	MOPS–glycerol–CAA	30	73	0.57	0.75

a Temperature (°C), doubling time (dt), growth rate (µ).

b *Frt* (FLP recognition target) indicates ‘scar’ sequence located downstream of the *rne* gene, which is formed upon removal of the drug resistant cassette by FLP recombinase (Datsenko and Wanner, 2000).

c CAA = casamino acids.

### Global slowdown in mRNA degradation

**Figure 1:**
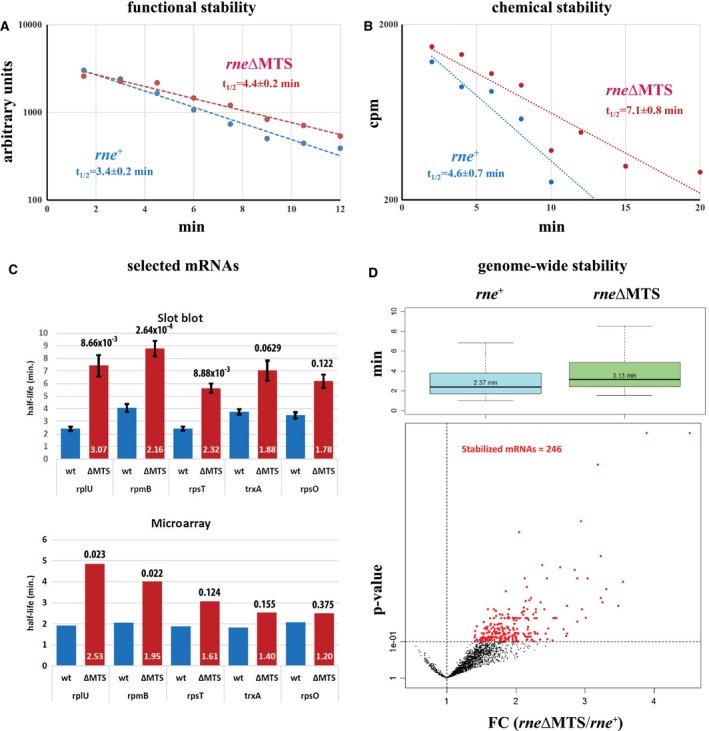
mRNA stability. A. and B. Functional and chemical stability of mRNA. Representative experiments with the* rne^+^* and *rne*∆MTS strains. Half‐lives are shown as the average and standard deviation of three replicates. C. Stability of selected mRNAs. Top panel. mRNA half‐lives determined by RNA slot blotting. Blue columns, *rne^+^* strain, red columns, *rne*ΔMTS strain. The numbers at the bottom of the red columns are the fold change in half‐life. The error bars represent standard deviations. The numbers at the top of the red columns are the associated *p*‐values. Bottom panel. mRNA half‐lives determined by microarray analysis. The numbers at the bottom of the red columns are the fold change in half‐life. The numbers at the top of the red columns are the associated *p*‐values. D. Genome‐wide measurement of mRNA stability by microarray analysis. Top panel. Box plots showing the distribution of 2038 mRNA half‐lives in the* rne* ^+^ and *rne∆MTS* strains. The mid‐line indicates the median half‐life. The difference in distribution between the two strains is highly significant (Kolmogorov–Smirnov test, *p*‐value <2.2 × 10^‐16^). Bottom panel. Volcano plot of *p*‐value versus fold‐change (FC) in mRNA half‐life. The red points represent 246 ORFs for which the associated *p*‐value was less than 0.1.

Since RNase E initiates mRNA degradation, we measured mRNA stability in the *rne∆MTS* strain using several different methods. First, we determined functional stability of mRNA by measuring protein synthesis rates by pulse labelling with ^35^S‐methionine after the addition of rifampicin. This experiment measures the functional stability of bulk mRNA as evidenced by the decrease in capacity to synthesize protein. The result of three independent measurements gave functional half‐lives of 3.4 ± 0.2 and 4.4 ± 0.2 min in the *rne^+^* and *rne∆*MTS strains respectively (Fig. 1A). Next, we determined chemical stability of bulk mRNA by pulse‐labelling RNA with ^3^H‐uridine and then measuring the rate of loss of radioactive RNA after addition of rifampicin. In this experiment, a proportion of the RNA, which corresponds to tRNA and rRNA, is stable. Degradation of the unstable proportion, which corresponds to mRNA, is measured after subtracting the radioactivity corresponding to the stable RNA. Three independent measurements gave mRNA chemical half‐lives of 4.6 ± 0.7 and 7.1 ± 0.8 min in the *rne^+^* and *rne∆MTS* strains respectively (Fig. 1B). These results show a significant slowdown of mRNA degradation in the *rne∆MTS* strain.

To validate the bulk mRNA decay measurements, we selected five mRNAs for analysis by slot blotting. In this experiment, the growth conditions were the same as in the chemical stability measurement (Fig. 1B). A pilot study showed that the *rplU*, *rpmB*, *rpsT*, *trxA* and *rpsO* mRNAs ran as discrete transcripts that could be detected in northern blots using ^32^P‐labelled oligonucleotides. Upon addition of rifampicin, these transcripts decayed monotonically with no detectable intermediates. We quantified mRNA stability by RNA slot blotting. After hybridization with mRNA‐specific oligonucleotides (Table S2), the blots were stripped and then hybridized with a probe specific to 23S rRNA. mRNA levels were normalized to 23S rRNA levels. Rates were measured by fitting the data to exponential decay curves. Half‐life, fold‐change, standard deviation and associated *p*‐values were calculated from the slot blot data (Fig. 1C upper panel, Table S3). Consistent with the bulk mRNA measurements, the rates of mRNA degradation in the *rne*ΔMTS strain were approximately 1.8 to 3.1‐fold slower than in the wild type control.

Next, we determined the rate of degradation of 2038 ORFs in the MG1655 background. M9‐glucose cultures at 37°C were grown to mid exponential phase and rifampicin was added to stop transcription. At times after addition of rifampicin, RNA was isolated. cDNA was prepared and hybridized to DNA microarrays to determine mRNA levels. After an initial normalization step between arrays, decay rates were determined by pooling the data from three independent replicates. The upper panel in Fig. 1D shows box‐plots of the distribution of mRNA half‐lives. The median half‐life was 2.37 min and 3.13 min in the *rne^+^* and *rne∆MTS* strains respectively. The difference in half‐life distribution is highly significant with a *p*‐value = 2.2 × 10^−16^ (Kolmogorov–Smirnov test). Median mRNA half‐lives in the range of 2–3 min are normal for growth in M9‐glucose at 37°C (Esquerre *et al.*, 2014). The lower panel in Fig. 1D is a Volcano plot of the relative change in mRNA half‐life in the *rne∆MTS* strain. The plot is highly skewed to increased half‐life showing that a large proportion of the messages are stabilized. The lower panel in Fig. 1C shows microarray results for the mRNAs whose half‐lives were determined by slot blotting. A comparison of the panels shows that the microarray and slot blot analyses are congruent. The rates of degradation in the upper panel are slower because the slot blot experiment was performed at 30°C. Slower rates of mRNA degradation and larger fold changes in half‐lives at 30°C likely contribute to the lower associated *p*‐values in the slot blot measurements.

By applying a cutoff of *p*‐value <0.1 (Volcano plot, Fig. 1D), 246 ORFs with a 1.5–4.5‐fold increase in half‐life (red dots) were selected. The stabilization of the 246 ORFs is not due to slower growth of the mutant strain since there was no significant difference in half‐lives of these ORFs in a previous analysis comparing stabilities at growth rates of µ = 0.40 h^−1^ vs. µ = 0.60 h^−1^ (Esquerre *et al.*, 2014). Functional analysis of the selected ORFs showed that 13 Gene Ontology (GO) terms were overrepresented. Enrichment includes mRNAs encoding proteins with inner membrane‐related functions such as lipid transport, protein insertion and proton transport (Fig. S1 and Table S4). However, messages encoding proteins with other functions such as translation, transcription and carbon metabolism were also enriched in the GO term analysis.

To test whether there was a general effect of the *rne∆MTS* mutation on the stability of specific classes of messages, we selected four groups of messages: 442 that encode IMPs (inner membrane proteins), 275 whose degradation is RppH‐dependent, 290 that are SRP‐dependent for translational insertion of encoded protein into the inner membrane, and 163 that encode proteins dependent on SecB for secretion. The control group for each analysis was the remainder of the 2038 ORFs that were not part of the test group. Although small differences in half‐life distribution were measured between the* rne^+^* and *rne∆MTS* strains, they were not significant (*p*‐values >0.01, Kolmogorov–Smirnov test) except in the case of the RppH‐dependent messages (5.6 × 10^−5^) (Fig. S2). The RppH‐dependent messages had shorter half‐lives compared to the control group in both the wild‐type and *rne∆MTS* strain showing that mRNA destabilization by RppH does not depend on RNase E membrane attachment. In the* rne∆MTS* strain, messages encoding IMPs or messages that are part of the SRP‐ and SecB‐dependent pathway were not differentially stabilized. Although there is an enrichment of a few messages encoding IMPs considering GO terms, we do not detect a global stabilization of messages encoding IMPs as reported previously (Moffitt *et al.*, 2016). This difference is not due to strain background since both measurements were in MG1655. Growth conditions could be a factor.

### Autoregulation of *rne* mRNA stability

**Figure 2:**
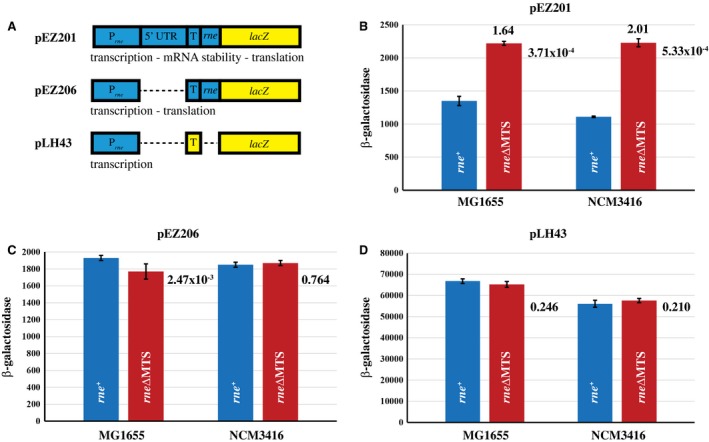
*rne*‐*lacZ* fusions. A. Schematic diagrams of *rne*‐*lacZ* fusions in pEZ201, pEZ206 and pLH43. Blue indicates *rne* sequences; yellow, *lacZ* sequences; P, promoter; 5′ UTR, 5′ untranslated region; T, translation initiation region; *lacZ*, β‐galactosidase coding sequence. The text under each construct indicates input signals that determine β‐galactosidase levels. In B–D. The error bars show standard deviations. The associated *p*‐values are indicated to the right of the red columns. B. pEZ201 β‐galactosidase levels. 1.64 and 2.01 indicate the fold change relative to the *rne^+^* strain. C. pEZ206 β‐galactosidase levels. D. pLH43 β‐galactosidase levels.

The *rne* mRNA is one of the 246 ORFs for which there is strong statistical support for stabilization in the *rne∆MTS* strain. There was a 1.5‐fold increase in half‐life and a 1.9‐fold increase in level (Table S5). To determine whether the increased mRNA level in the transcriptome analysis results in higher levels of cRNase E synthesis, we used *rne*‐*lacZ* fusions that measure effects on transcription, translation and mRNA stability (Fig. 2A). pEZ201 is a fusion of *rne* expression signals and the beginning of the *rne* coding sequence to the *lacZ* coding sequence (Jain and Belasco, 1995). pEZ206 has a deletion of most of the 5′ UTR that results in disruption of the autoregulation of RNase E synthesis. pLH43 is a fusion of *rne* transcription signals to the *lacZ* 5′ leader and coding sequence.

We measured β‐galactosidase levels under conditions used in the genome‐wide analysis of mRNA stability. In the MG1655 and NCM3416 isogenic pairs, β‐galactosidase levels in the *rne∆MTS* strain were 1.64‐fold and 2.01‐fold higher respectively (Fig. 2B). Increased β‐galactosidase levels therefore correlate with increased mRNA levels in the transcriptome analysis. This effect is due to the autoregulation signals as evidenced by little or no difference in the levels of expression with pEZ206 and pLH43 (Fig. 2C and D). Similar results were obtained with growth in MOPS–glycerol–casamino acids at 30°C and LB at 37°C (Table S6). In MOPS–glycerol–casamino acids at 30°C, we also observed an effect of the *rne∆MTS* mutation on transcription, which was not further investigated. These results show that cRNase E synthesis in the *rne∆MTS* strain is higher than in the wild‐type strain under all conditions tested. Since increased levels of mRNA are due to autoregulation, these results suggest that higher levels of cRNase E synthesis are a response to an increased demand for ribonuclease activity.

### RNase E stability

**Figure 3:**
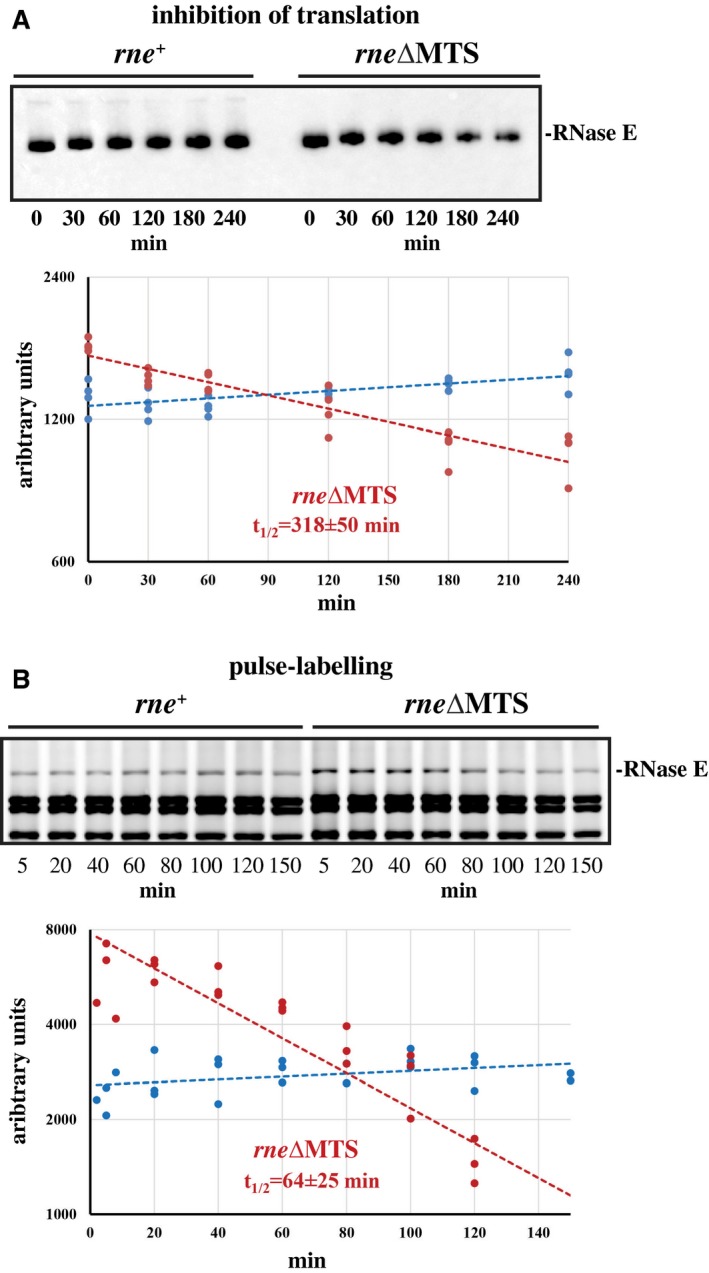
RNase E stability. A. Inhibition of translation by chloramphenicol. Top panel, representative western blot. Bottom panel, quantification of cRNase E stability. B. Pulse labelling with ^35^S‐methionine. Top panel, representative SDS‐PAGE gel. Bottom panel, quantification of cRNase E stability. In parts A and B, the blue line corresponds to the *rne^+^* strain; the red line to *rne*∆MTS. The mean half‐life and standard deviation were calculated from three (A) or four (B) replicates

Considering the results of the previous section, we expected that the level of cRNase E would be higher than the wild‐type control. This, however, was not the case. Quantitative western blotting showed that RNase E levels in the *rne^+^* and *rne∆MTS* strains are the same regardless of strain background or growth condition (Fig. S3 and Table S7). The apparent contradiction between the *rne‐lacZ* fusions and protein levels measured by western blotting suggested that cRNase E is an unstable protein. We therefore determined protein stability by inhibiting translation with chloramphenicol and measuring protein levels by western blotting. Figure 3A, top panel, shows a representative western blot. The bottom panel shows the quantification of three independent experiments. The higher level of cRNase E vs. RNase E at time 0 is due to a slightly higher OD_600_ when chloramphenicol was added. There is a small increase in level in the *rne^+^* strain, which is due to leaky growth over the 4‐h time course. The results of three independent experiments showed that cRNase E is unstable with a half‐life of 318 ± 50 min.

Although the chloramphenicol experiment validates the instability of cRNase E, the rate of decay is too slow to account for the level of increased expression of the *rne∆MTS* allele in exponentially growing cells. We therefore measured RNase E stability by pulse chase with ^35^S‐methionine (Fig. 3B). In this experiment, cells were labelled at low density and growth continued after the chase with a large excess of cold methionine. During the 150‐min time course, equal volumes of culture were sampled to measure the level of radioactive RNase E. In the wild‐type control, there is a small increase in RNase E. Since the protocol involved TCA precipitation, this increase could be due to slightly better yields as the density of the culture increased. Note that there is nearly twofold more newly synthesized cRNase E (red dots) than RNase E (blue dots) at the zero time point, which is direct evidence for a higher rate of synthesis. The results of four independent experiments showed that cRNase E was unstable with a half‐life of 64 ± 25 min. These results show that the rate of degradation of cRNase E depends on translation and cell growth. The instability of cRNase E measured in growing cultures is compatible with the increased synthesis of cRNase E, which compensates for protein instability.

### RNA degradosome assembly

**Figure 4:**
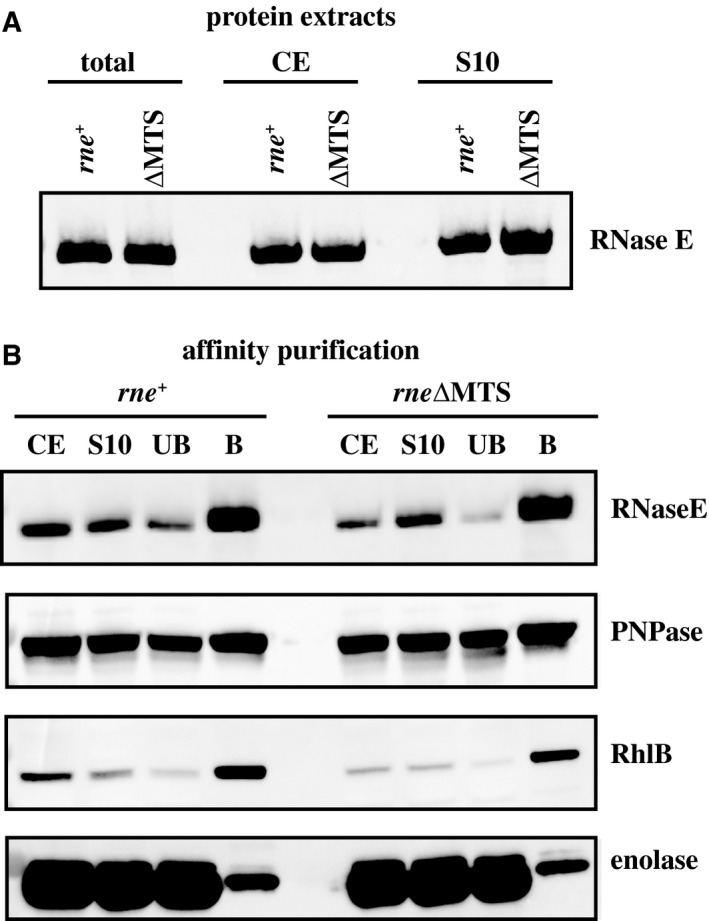
RNA degradosome assembly. A. Protein extracts. Western blotting of total protein (before cell lysis), crude extract (CE, after cell lysis) and 10,000 g supernatant (S10). B. Affinity purification. The S10 fraction was applied to beads with anti‐FLAG antibody. After a wash to remove unbound (UB) protein, the bound (B) protein was eluted by competition with FLAG peptide. Each panel shows western blotting of the CE, S10, UB and B fractions with anti‐FLAG antibody (RNase E) or polyclonal antisera against PNPase and enolase. For reasons of specificity, an RhlB‐GFP fusion was detected using anti‐GFP antibody. Previous work showed that the RhlB‐GFP fusion associates with RNase E and has helicase activity (Strahl *et al.*, 2015).

We next examined the possibility that cRNase E instability is due to a defect in RNA degradosome assembly. We analyzed RNA degradosome composition by affinity purification of RNase E. Western blot analysis of total protein, crude protein extract (CE) and clarified supernatant (S10) showed that RNase E and cRNase E were efficiently solubilized (Fig. 4A). Incubation of the S10 fraction with anti‐FLAG beads, washing and elution with FLAG peptide yielded the unbound (UB) and bound (B) fractions. Western blotting showed that RNase E and cRNase E co‐purified with PNPase, RhlB and enolase (Fig. 4B). There was no detectable difference in the level of the proteins associated with cRNase E and RNase E. cRNase E instability is therefore not a consequence of a defect in RNA degradosome assembly.

### RNase E activity

**Figure 5:**
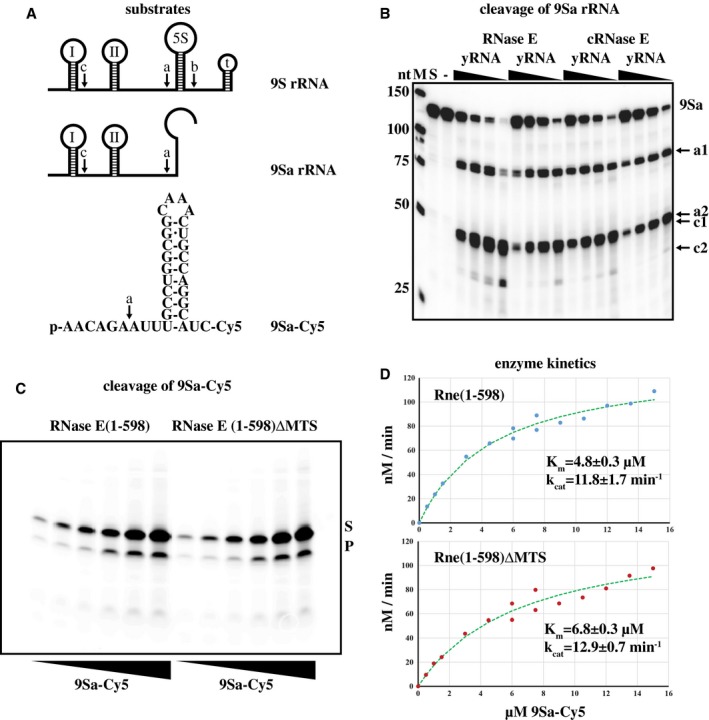
RNase E activity. A. Map of 9S rRNA and a truncated derivative, 9Sa, which was used as substrate in the assay shown in panel B. a, b and c show RNase E cleavage sites (see Carpousis *et al.*, 2001). 9Sa‐Cy5 is a synthetic 35‐nt derivative of 9S rRNA containing a 5′ monophosphate, the a‐cleavage site, a stem‐loop that corresponds to a fragment of 5S rRNA, and a Cy5 group at the 3′ end. B. RNase E and cRNase E activity. Radiolabeled 9Sa RNA substrate was digested with two different concentrations of enzyme in the presence of 250, 125, 75 or 25 µg yeast RNA as competitor. The products were separated on a denaturing polyacrylamide gel and visualized by phosphorimaging. M, markers; S, substrate; ‐, mock digestion in which RNase E was omitted. The cleavage products are indicated on the right. C. Representative gel showing the cleavage of the 9S‐Cy5 substrate by RNase E(1‐598) and RNase E(1‐598)∆MTS. The 35 nt substrate (S) and 29 nt product (P) were visualized by fluoroimaging after separation on a native 20% TBE gel. D. Kinetic analysis of the RNase E(1‐598) and RNase E(1‐598)*∆*MTS. Initial velocities were determined at two different concentrations of enzyme. Substrate ranging from 0.5 to 7.5 µM was digested with 10 nM enzyme; substrate ranging from 6.0 to 15 µM with 20 nM enzyme. The data were fitted to the Michaelis–Menten equation (Experimental procedures). The K_m_ and k_cat_ are the mean and standard deviation of three replicates.

Autoregulation of *rne∆MTS* mRNA stability resulting in levels of cRNase E equal to RNase E suggests that the catalytic activity of cRNase E and RNase E is comparable. We measured the activity of purified cRNase E and RNase E *in vitro*. We first tested radiolabeled 9Sa substrate, which is a fragment of 9S rRNA (Fig. 5A). The enzymes were tested at two concentrations in the presence of a range of cold competitor RNA (Fig. 5B). In this assay, the principal cleavage is at the a‐site, yielding 81 and 47 nt fragments (a1 and a2, respectively) with a slower secondary cleavage at the c‐site converting the 81 nt fragment to 45 and 36 nt fragments (c1 and c2 respectively). There was no significant difference in the activity of RNase E and cRNase E based on visual inspection of the image in Fig. 5B.

To measure kinetic constants, we developed an assay using Rne(1‐598), which is a fragment of RNase E that contains the catalytic domain, MTS and a C‐terminal HIS tag. We also tested Rne(1‐598)*∆*MTS, a variant in which 15 residues corresponding to the MTS were deleted. RNase E cleavage of the 35 nt 9Sa‐Cy5 substrate (Fig. 5A), which results in a 29 nt product, was monitored by fluoroimaging after native gel electrophoresis (Fig. 5C). Figure 5D shows a Michaelis–Menten analysis of the activity of Rne(1‐598) and Rne(1‐598)*∆*MTS. The results of three replicates showed no difference in k_cat_, but a small difference in K_m_ (4.8 ± 0.3 and 6.8 ± 0.3 µM, Rne(1‐598) and Rne(1‐598)*∆*MTS respectively). The rate of cleavage of the *∆*MTS enzyme is 91% of the wild‐type enzyme at a substrate concentration of 4.8 µM. The increased synthesis of RNase E via autoregulation could include a contribution from lower activity of cRNase E due to the difference in K_m_. However, the experimental results strongly suggest that cRNase E instability is the major contributor to increased expression.

### CsrB/C stability

**Figure 6:**
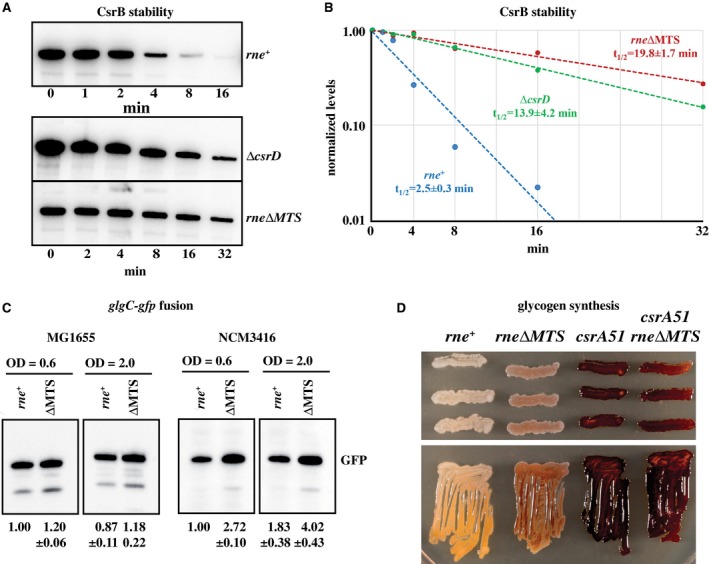
Carbon storage regulation. A. Representative northern blots of CsrB levels after rifampicin treatment in the *rne^+^*, ∆*csrD* and *rne*∆MTS strains. B. Quantification of CsrB decay rates. The half‐lives are the mean and standard deviation of three replicates. C. Effect of the *rne∆MTS* allele on the expression of *glgC*. Strains harboring pLH40 with a *glgC*‐*gfp* fusion were grown on LB at 37°C to the indicated OD_600_. Expression levels were determined by western blotting using anti‐GFP antibody. GFP levels relative to the *rne* ^+^ control are shown at the bottom of each panel as the mean and standard deviation of at least three replicates. D. Glycogen detection in the MG1655 background by staining with iodine vapor. The plates were streaked or patched from single colonies onto LB agar plates with 2% glucose and incubated at 37°C overnight. Iodine exposure was for 2 min at 37°C.

Since membrane‐associated CsrD regulates RNase E‐dependent degradation of CsrB/C (Suzuki *et al.*, 2006), we asked if the attachment of RNase E to the inner membrane is necessary for CsrB/C degradation. Figure 6A shows northern blots in which CsrB stability was measured in the MG1655 background in M9‐glucose medium at 37°C. The results of three independent measurements of half‐life of CsrB in the *rne^+^* strain was 2.5 ± 0.3 min (Fig. 6B). There were eightfold increases in the half‐life of CsrB in the *rne∆MTS* strain (19.8 ± 1.7 min). The knockout of *csrD* (∆*csrD*), which is shown as a control, had an intermediate effect (13.9 ± 4.2 min). The large stabilization of CsrB in the *rne∆MTS* strain does not correlate with a large increase in steady‐state level. As has already been shown for the *csrD* knockout (Suzuki *et al.*, 2006), this effect is likely due to feedback regulation of transcription. CsrC was also stabilized in the* rne∆MTS* strain (Fig. S4). Similar results for CsrB/C were obtained with growth on LB medium and in the NCM3416 background. We conclude that RNase E attachment to the inner cytoplasmic membrane is necessary for the degradation of CsrB/C.

A hallmark of carbon storage regulation is inhibition of glycogen synthesis during growth on rich media. We therefore examined the effect of the *rne∆MTS* mutation on the levels of the *glgC* mRNA, which encodes a glycogen synthesis enzyme (Baker *et al.*, 2002). The *glgC* mRNA is a target of translational repression by CsrA. We used a fusion of the 5′ transcription and translation signals of *glgC* to the *gfp* coding sequence and measured GFP levels by western blotting. In the MG1655 background, there is a small but a significant increase in the level of GFP in the *rne∆MTS* strain in both exponentially growing cells as well as upon entry into stationary phase (Fig. 6C). This effect is more pronounced in the NCM3416 background. These results strongly suggest that the *rne∆MTS* mutation results in lower levels of free CsrA. We therefore made a qualitative plate assay in which glycogen was stained by exposure to iodine (Fig. 6D). This result shows that glycogen synthesis is leaky in the *rne∆MTS* strain. The *csrA51* mutation, which results in de‐repression of glycogen synthesis, is shown as a control.

### Stability of ribosome‐free transcripts

**Figure 7:**
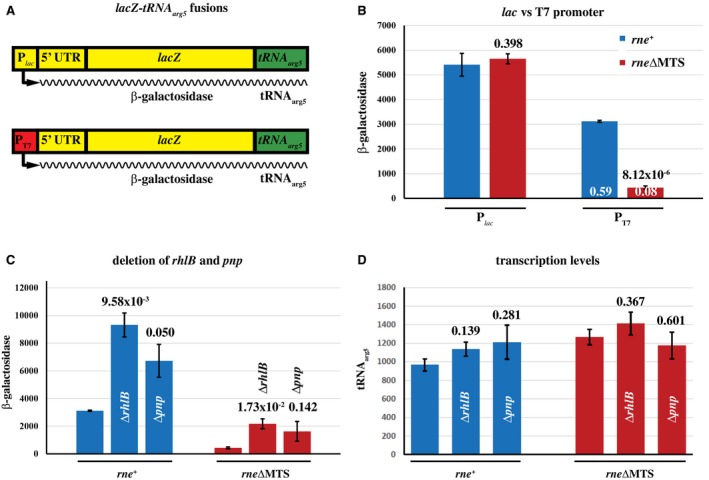
Stability of ribosome‐free transcripts. A. Schematic diagram of single copy chromosomal constructs of the *lacZ*‐*tRNA_arg5_* fusion under the control of either the P*_lac_* or P*_T7_* promoters. Yellow, *lac* sequences; green, *tRNA_arg5_* sequences; red, bacteriophage T7 sequences. In panels B.‐D. The error bars show standard deviations. The numbers above the columns are the associated *p*‐values. B. β–galactosidase levels with the P*_lac_* and the P*_T7_* constructs. The numbers at the bottom of the columns indicate the level of β–galactosidase relative to the P*_lac_* construct. C. β–galactosidase levels in strains in which the genes encoding RhlB and PNPase were disrupted. The numbers at the bottom of the columns indicate the level of β–galactosidase relative to the *rne^+^* or *rne*∆*MTS* strain. D. Quantification of the tRNA_arg5_ levels. RNA for northern blotting (Fig. S5) was made from the same cultures that were used to determine β–galactosidase levels. The numbers above the columns are the associated *p*‐values.

The coupling of transcription to translation is necessary for mRNA stability as evidenced by the instability of transcripts synthesized by bacteriophage T7 RNA polymerase, which elongates transcripts eightfold faster than the *E. coli* RNA polymerase (Lopez *et al.*, 1994; Iost and Dreyfus, 1995). Since elongation by T7 RNA polymerase is faster than translation, ribosome‐free mRNA is cleaved by RNase E resulting in a lower yield of protein per message. In view of the global slowdown of mRNA degradation in the* rne∆MTS* strain, we asked whether there was also a slowdown in the inactivation of ribosome‐free mRNA. We used a synthetic construct in which *lacZ* mRNA is transcribed from a bacteriophage T7 promoter (Lopez *et al.*, 1994; Iost and Dreyfus, 1995; Lopez *et al.*, 1999; Leroy *et al.*, 2002). As shown in Fig. 7A, a message consisting of the 5′ UTR and coding sequence of *lacZ* fused to a tRNA_arg5_ sequence is transcribed by T7 RNA polymerase from a single‐copy gene in the chromosome. The tRNA portion of the transcript is processed to a stable product that is used to measure the level of transcription. This construct has the advantage of obviating the need to measure mRNA half‐lives to distinguish between transcription and degradation effects. The same construct, but with a *lac* promoter driving transcription by *E. coli* RNA polymerase, is a control. The resulting transcripts are identical. The yield of protein per mRNA is measured by normalizing β‐galactosidase levels to tRNA_arg5 _levels.

As expected, there is no significant difference in the levels of β‐galactosidase with the P*_lac_* construct in the *rne^+^* and *rne∆MTS* strains (Fig. 7B, Table S8). Transcription with bacteriophage T7 RNA polymerase results in a lower level of β–galactosidase in the* rne^+^* strain as expected, whereas there is a much larger decrease in the *rne∆MTS* strain (0.59 and 0.08, respectively, relative to P*_lac_* construct). Since the level of transcription, as measured by probing for the 5′ tRNA, is comparable in the *rne^+^* and* rne∆MTS* strains (Figs 7D and S5), this result shows that there is a large decrease in the yield of protein per mRNA in the *rne∆MTS* strain. Fig. 7C shows an increase in the yield of protein per mRNA when the genes encoding RhlB and PNPase are disrupted. The *rne^+^* strain is shown as a control (Khemici *et al.*, 2005). This result shows that RhlB and PNPase as part of the cytoplasmic RNA degradosome participate in the inactivation of the *lacZ* mRNA. These results contrast strikingly with the global slowdown in mRNA decay in the *rne∆MTS* strain and show that membrane attachment protects ribosome‐free transcripts from inactivation by the RNA degradosome.

## Discussion

### mRNA degradation

We measured the half‐lives of 2038 mRNAs in the *rne^+^* and* rne∆MTS* showing that there is a global slowdown in mRNA degradation. Under our growth conditions, there was no correlation between mRNA stabilization and the function or cellular location of encoded proteins. Our results differ from previous work showing that mRNAs encoding IMPs are destabilized by a mechanism that requires the attachment of RNase E to the inner membrane. However, we measured stability at 37°C in M9‐glucose medium, whereas the previous work was at 32°C in LB medium. Besides the difference in temperature, there is a major remodeling of intermediary metabolism between growth in M9‐glucose, which is glycolytic, and growth in LB, which is gluconeogenic. If repression of IMP synthesis by destabilization of mRNA is a regulated process, then our results suggest that expression is de‐repressed in M9‐glucose at 37°C. To our knowledge, there is no work in the literature describing regulated stability of IMP mRNAs. Identifying regulatory factors and elucidating the mechanism of selective destabilization of the IMP mRNAs is a potentially fruitful area for future research.

The global slowdown of mRNA degradation in the *rne∆MTS* strain is striking because our results show that the level of cRNase E is the same as wild‐type RNase E. Since autoregulation of *rne∆MTS* expression compensates for the instability of cRNase E, the slowdown in mRNA degradation is not due to a decrease in RNase E level. Furthermore, *in vitro* kinetic assays performed here show that the ribonuclease activity of RNase E and cRNase E are comparable. In previous *in vitro* work, we showed that the addition of protein‐free multilaminar phospholipid vesicles or Triton X‐100 had no effect on cleavage of radioactively labelled 9Sa substrate (Khemici *et al.*, 2008). Taken together, these results show that association with phospholipid membrane does not affect RNase E activity. We have evidence that there is an accumulation of small RNA fragments in the* rne∆MTS* strain (unpublished work), suggesting a slowdown in the turnover of RNA degradation intermediates. The origin of these fragments is currently under investigation.

### CsrB/C stability

We have shown that attachment of RNase E to the inner cytoplasmic membrane is necessary for turnover of CsrB/C. CsrD‐dependent degradation depends on an RNase E cleavage in the 3′ end of CsrB (Suzuki *et al.*, 2006; Leng *et al.*, 2016; Vakulskas *et al.*, 2016). CsrD is activated by an interaction with EIIA^glc^, which is a component of the membrane‐associated glucose‐specific PTS system. The work reported here shows that membrane attachment of RNase E is necessary for regulated degradation of CsrB/C. Co‐localization of RNase E, CsrD and EIIA^glc^ could facilitate their interaction by increasing local concentration on the two‐dimensional surface of the inner membrane and constraining their orientations to increase the probability of productive interactions.

We have tested the stability of a library of sRNAs in the *rne∆MTS* strain and found that GcvB is stabilized compared to the *rne^+^* strain (unpublished results). A target of GcvB is *csgD* mRNA, which is a central regulator of biofilm formation (Jorgensen *et al.*, 2012). Recent work has shown that *csgD* mRNA degradation is also RNase E‐dependent (Andreassen *et al.*, 2018). By analogy to CsrB/C, GcvB turnover could require interaction with a regulatory factor on the inner membrane. Proteomics surveys have shown that there are over 700 proteins in *E. coli* that are either directly or indirectly associated with the inner membrane, suggesting the potential for numerous interactions with RNase E in the posttranscriptional control of gene expression (Papanastasiou *et al.*, 2013; Papanastasiou *et al.*, 2016).

### Stability of ribosome‐free transcripts

Our results show that the yield of β‐galactosidase per P*_T7_*‐*lacZ* mRNA is 12.5‐fold lower in the *rne∆MTS* strain compared to P*_lac_*‐*lacZ* mRNA. This difference is due to a large increase in the instability of ribosome‐free mRNA. Super‐resolution imagining of RNase E in live cells has shown that there is little or no RNase E localized to the cytoplasm or the nucleoid, whereas cRNase E is distributed uniformly throughout the cell (Moffitt *et al.*, 2016). We propose that interactions between nascent transcripts and membrane‐associated RNase E are infrequent due to the spatial separation of transcription in the nucleoid from RNase E attached to the inner membrane. In contrast, in the *rne∆MTS* strain, cRNase E has direct access to ribosome‐free transcripts thereby increasing the probability of inactivation. A corollary of this interpretation is that coupled transcription‐translation by *E. coli* RNA polymerase protects newly synthesized mRNA from premature inactivation by cRNase E.

Genetic work with suppressor mutations has suggested a link between Rho‐dependent transcription termination and RNase E although the molecular mechanism is not understood (Anupama *et al.*, 2011). The suppressor work relied on an RNase E(1‐490) mutant, which is cytoplasmic. Rho acts on ribosome‐free transcripts by translocating on RNA to ‘catch’ the RNA polymerase and promote termination (Boudvillain *et al.*, 2013). P*_T7_* transcripts escape Rho‐dependent termination because elongation by T7 RNA polymerase is faster than Rho translocation. Our results clarify the relationship between RNase E attached to the inner membrane and Rho in the nucleoid. Cytoplasmic mutants of RNase E could interfere with termination by cleaving ribosome‐free nascent transcripts before Rho has a chance to act.

### Stability of RNase E *in vitro*

Our determination that cRNase E and RNase E have comparable activities differs from work by other investigators who used a fragment of RNase E corresponding to residues 1‐499 (Murashko *et al.*, 2012). Analytical ultracentrifugation and X‐ray crystallography have shown that RNase E is a tetramer (Callaghan *et al.*, 2004; Callaghan *et al.*, 2005). Residues 500–510 form an α‐helix with a highly conserved tyrosine at position 500, which is buried in an interface necessary for the quaternary structure of the enzyme (see Protein Data Bank, 2BX2; (Bouvier and Carpousis, 2011)). Our *in vitro* work used either full‐length RNase E or a fragment of RNase E extending from residues 1‐598. In our hands, the fragment is stable and fully active. Deletion of the MTS has a small effect on the K_m _of the enzyme, but not the k_cat_. We therefore believe that truncation of RNase E at residue 499 results in disruption of the tetrameric structure of RNase E and loss of enzyme stability and ribonuclease activity.

### Stability of RNase E *in vivo*

The instability of cRNase E *in vivo* suggests that RNase E attached to the inner membrane is protected from degradation either by shielding sites that are susceptible to proteases or by sequestering the enzyme from cytoplasmic proteases. Instability could have important regulatory consequences. Disruption of the MTS, for example, by proteolysis or chemical modification, could trigger degradation of RNase E as part of a response that downregulates ribonuclease activity. Recent work appears to support this hypothesis. Upon transition from aerobic to anaerobic conditions, RNase E moves to the cytoplasm and is degraded (Murashko and Lin‐Chao, 2017). The biological consequence of degrading RNase E could be to inhibit RNA processing and degradation during remodeling of gene expression. Elucidating mechanisms that release RNase E from the inner cytoplasmic membrane could be an important avenue for future work on the control of ribonuclease activity in *E. coli*.

### Concluding remark

A major finding of this work is that detachment of RNase E from the inner cytoplasmic membrane results in a global slowdown of mRNA degradation. Video imaging (TIRFm) has shown that membrane associated RNase E rapidly diffuses on the inner membrane and forms short‐lived foci (Strahl *et al.*, 2015). Super‐resolution microscopy (3D STORM) has shown that RNase E is uniformly distributed on the inner cytoplasmic membrane (Moffitt *et al.*, 2016). These seemingly contradictory results are in fact congruent since TIRFm video is real time imaging on the scale of seconds, whereas super‐resolution imaging is a reconstruction of data collected over several minutes. These results show that RNase E foci formation is highly dynamic, but when averaged over time, RNase E is uniformly distributed on the inner membrane.

Experimental evidence strongly suggests that RNase E foci are centers of RNA processing and degradation (Strahl *et al.*, 2015). The brightness of the foci shows that they contain multiple copies of RNase E. Recent work with *Caulobacter crescentus* RNase E has shown that the intrinsically unstructured noncatalytic region promotes formation of ribonucleoprotein (RNP) condensates that could affect catalytic efficiency and substrate selectivity of the RNA degradosome (Al‐Husini *et al.*, 2018). These condensates are similar to eukaryotic P‐bodies and stress granules (Banani *et al.*, 2016; Hubstenberger *et al.*, 2017). Transplantation of the noncatalytic region of *E. coli* RNase E to the *Caulobacter* enzyme also promotes the formation of RNP condensates. Since cRNase E in our study does not appear to form bright foci (Strahl *et al.*, 2015), we propose that detachment of the *E. coli* enzyme from the inner membrane interferes with RNP condensation and thereby slows mRNA degradation. Research on the dynamics, composition and activity of membrane associated RNP condensates in *E. coli* should help to better understand their role in mRNA degradation.

## Experimental procedures

### Strains

Strains are listed in Table S1. To make *E. coli* constructs with a single copy of the *rne∆MTS* allele, λ Red recombineering was performed as described (Strahl *et al.*, 2015). After allele substitution into the chromosome using a *cat* cassette, the construct was genetically purified by bacteriophage phage P1 transduction and the *cat* cassette was removed using FLP recombinase resulting in an *frt* (FLP recognition target) scar (Datsenko and Wanner, 2000). All constructs were validated by sequencing PCR products amplified from chromosomal DNA.

Strains LHS393 and LHS395 were constructed using a PCR product from TM338 corresponding to sequences encoding the C‐terminus of RNase E, a FLAG epitope and the *cat* gene. The Δ*glgC* mutants were obtained by P1 transduction from the Keio strain collection (Baba *et al.*, 2006) into LHS393 and LHS395.

The MGM strains are strictly isogenic with the ENS133 and ENS134 parent strains. λ Red recombineering into the ENS134 background was employed as a first step since there is approximately 1% sequence heterogeneity between *E. coli* K12 (NCM3416) and B (BL21) strains.

### Plasmids

Plasmids are listed in Table S1. pLH40, pLH43, pLP56‐2 and pLP57‐2 were made by InFusion cloning (Clonetech). pLH40 is a derivative of pXG10 (Urban and Vogel, 2007) in which *glgC* expression signals (+1 of transcription to the 15th codon) were fused to the *gfp* coding sequence (Baker *et al.*, 2002). pLH43 is a derivative of pSAB11 (Ait‐Bara and Carpousis, 2010) in which *rne* expression signals up to the +1 transcription initiation site were fused to the* lacZ* translation initiation region (−22 to −1) and coding sequence. pLP56‐2 is a derivative of pET21b‐*rne* in which the coding sequence from residues 599 to 1061 was deleted. pLP57‐2 is a derivative of pLP56‐2 in which the MTS sequence (residues 567–582) was deleted.

### Culture media

M9‐glucose and MOPS minimal media were prepared as described (Neidhardt *et al.*, 1974; Esquerre *et al.*, 2014). Casamino acids and glycerol were added to final concentrations of 0.2% and 0.4% respectively. Cultures were grown with shaking at 180 rpm.

### Functional stability of mRNA

Functional stability of mRNA was measured as described with minor modifications (Lopez *et al.*, 1999). Cultures in MOPS medium supplemented with glycerol and a mix of l‐amino acids (50 µg ml^−1^ A, R, N, D, C, E, G, H, I, L, K, P, S, T, V, Q) were grown at 30°C to mid exponential phase. After addition of rifampicin (500 µg ml^−1^), aliquots were withdrawn and proteins were pulse labelled with 5 µCi of ^35^S‐methionine. The radioactive methionine was diluted with cold methionine (0.075 µg ml^−1^ final concentration) so that less than 15% of the ^35^S‐methionine was incorporated thus ensuring measurement of rates of protein synthesis. After separation by SDS‐PAGE, incorporated radioactivity was quantified by phosphorimaging.

### Chemical stability of mRNA

Chemical degradation of mRNA was determined by measuring the loss of ^3^H‐uridine labelled RNA at times after addition of rifampicin. The temperature and growth medium was the same as in the measurement of inactivation of mRNA except that casamino acids were used instead of a mixture of l‐amino acids. A culture (10 ml) was labelled for 1 min with 20 µCi ^3^H‐uridine and then chased with the addition of rifampicin, naladixic acid and uridine (500, 20 and 200 µg ml^−1^ respectively). Aliquots were withdrawn, precipitated with 5% TCA, filtered on Whatman glass microfiber filters, washed with ethanol and dried. Radioactivity was measured by liquid scintillation counting. The level of stable RNA was estimated by taking the average of counts at 30, 40 and 50 min, which was then subtracted from the earlier time points.

### Slot blots

Strains were cultured and treated with rifampicin as described for the chemical stability measurements. Samples (20 ml) were withdrawn and quenched with 4 ml of a solution of 5% phenol in ethanol. Cells were pelleted by centrifugation in an Eppendorf S‐4‐72 rotor at 4°C for 20 min. After suspension in 1.5 ml of Tri Reagent (MRC) using a MultiTherm agitator (37°C, 550 rpm, 10 min), cell debris was removed by centrifugation (20,000 g, 10 min, room temperature). An equal volume of ethanol was added to the supernatant and total RNA was prepared using a Direct‐zol^TM^, RNA MiniPrep Plus kit (Zymo Research) following the manufacturer’s instructions. RNA was eluted in 80 µl of water. Concentration and purity were determined using a NanoDrop^TM^ spectrophotometer. RNA (2.5–4.0 µg) was heated at 65°C for 5 min in a 75 µl reaction containing 50% formamide, 2.5 M formaldehyde, 10 mM sodium MOPS, pH 7.0, 4 mM NaCl, 0.5 mM EDTA, 0.02% XC, 0.02% BPB and then vacuum blotted onto Hybond^TM^‐XL filters (Amersham) using a P648 slot blot manifold (Amersham) following manufacturer’s instructions. After UV cross‐linking (Hoefer, UVC 500, 120,000 µJ/cm^2^), the blots were hybridized with 5′‐^32^P‐DNA probes (Table S2). The *rpsT*, *trxA* and *rpsO* messages were hybridized with two probes to increase sensitivity. For normalization, the blots were stripped and then hybridized with a probe specific to 23S rRNA.

### Genome‐wide measurement of mRNA half‐lives

Genome‐wide mRNA half‐lives were measured as described (Esquerre *et al.*, 2014). Wild‐type and *rne∆MTS* strains (MG1655) were grown in M9‐glucose medium at 37°C to mid‐exponential phase and rifampicin was added to stop transcription. At times ranging from 0.5 to 11 min after addition of rifampicin, RNA was isolated, cDNA was prepared and hybridized to DNA microarrays to determine mRNA levels. A normalization between arrays according to an invariant probeset intensities was performed. For each strain, a set of probes in the background for which the ranks were roughly invariant across all arrays was selected. The median value of the invariant probeset intensities in each strain was used as a scaling factor for normalization between strains. After normalization, the intensity of a transcript was calculated by a RMA‐summarization procedure (Irizarry *et al.*, 2003) within each strain. In each array, transcript‐specific intensity was computed as the median value of the 16 targeting probe intensities. Raw and processed microarrays data were deposited on Gene Expression Omnibus data repository and are accessible through GEO Series accession number GSE118058 (https://www.ncbi.nlm.nih.gov/geo/query/acc.cgi?acc=GSE118058).

The linear regression coefficient, k, of ln(mRNA) versus time and its associated coefficient of variation (standard error of slope/estimation of slope) were calculated for each mRNA species after pooling the data from three independent replicates. The determination of k was considered as reliable only if the associated coefficient of variation was below 30%. The linear regression coefficient k corresponding to the degradation rate constant is inversely proportional to the mRNA half‐life, t_1/2 _(k = ln2/t_1/2_). The statistical significance of differences in half‐life was evaluated using the probability value of interaction between time and strains in a global model of linear regression. A statistical threshold of 10% was used for adjusted *p*‐values by the ‘BH’ FDR method (Benjamini and Hochberg, 1995).

Functional categories enriched in transcript subgroups were determined using hypergeometric test of data using the Biological Process of Gene Ontology annotation database (GO project; http://www.geneontology.org/). Enrichment significance was set with a cut‐off of 5% for the associated *p*‐value.

### β‐galactosidase assays

For *rne‐lacZ* expression, wild‐type and *rne∆MTS* strains transformed with pEZ201, pEZ206 or pLH43 were grown on M9‐glucose or LB at 37°C or on MOPS–glycerol–casamino acids at 30°C to an OD_600_ of 0.3. The strains with pEZ201 and pEZ206 were grown in the presence of 100 µg ml^−1^ ampicillin; pLH43 in the presence of 40 µg ml^−1^ spectinomycin. β‐galactosidase assays were performed as described (Khemici *et al.*, 2005).

For T7‐*lacZ* expression, the MGM strains were grown on MOPS–glycerol–casamino acids at 30°C to an OD_600_ of 0.3. β‐galactosidase activity was measured as described (Zhang and Bremer, 1995). For the determination of T7‐*lacZ* transcription levels (Fig. 7D), RNA was prepared from the same cultures that were used to make the β‐galactosidase assays. Total RNA (5 µg) was separated on an agarose gel. tRNA*_arg5_* levels were quantified by northern blots probed with ^32^P‐labelled oligonucleotides (Table S2). After hybridization with the tRNA*_arg5_* probe, the blot was stripped and hybridized with a 16S rRNA probe, which served as a loading control.

### RNase E stability

LHS393 and LHS395 strains are an isogenic pair corresponding to the *rne^+^* and *rne∆MTS* strains, respectively, but with the addition of a FLAG tag at the C‐terminal end of RNase E (Table S1). Cultures were grown on LB medium at 37°C to an OD_600_ = 0.6. Chloramphenicol (25 µg ml^−1^) was added to inhibit protein synthesis. At times after the addition of chloramphenicol, cells were collected, lysed and the level of RNase E was determined by western blotting using anti‐FLAG antibody and ECL detection.

For pulse‐labelling of RNase E, the same strains were cultured in MOPS medium supplemented with glycerol (0.4%) and a mix of l‐amino acids plus 0.1 µg ml^−1^ methionine (see **Functional stability of mRNA**). Overnight cultures were diluted into fresh medium and grown at 37°C to an OD_600_ = 0.4. 10 ml of culture was pulse‐labelled by addition of 500 µCi ^35^S‐methionine, then chased by addition of cold methionine (2 mg ml^−1^). At times after addition of the chase, 0.5 ml of culture was removed and added to 70 µl of cold TCA. Precipitated protein was rinsed with acetone, suspended in SDS loading buffer and separated by 7.5% SDS‐PAGE. Gels were soaked in 40% ethanol, 10% acetic acid, 2% glycerol, dried under vacuum at 80°C and exposed on a Phosphorimager screen overnight. RNase E levels were quantified using ImageQuant software.

### RNA degradosome assembly

LHS420 and LHS 422 differ from the LHS393 and LHS395 isogenic strains by a GFP fusion to the C‐terminus of RhlB (Table S1). RhlB‐GFP has been shown previously to be associated with RNase E as part of the RNA degradosome (Strahl *et al.*, 2015). GFP was used as a tag for western blotting. Cultures were grown in LB at 37°C to an OD_600_ = 0.6. Preparation of protein extracts, affinity purification of FLAG‐tagged RNase E and western blotting were performed as described (Khemici *et al.*, 2008; Ait‐Bara and Carpousis, 2010). Briefly, the following modifications were made for affinity purification: 1 mM EDTA was used throughout; anti‐FLAG M2 agarose was purchased from Sigma. Proteins were separated on 4%–12% NuPAGE gels with MOPS SDS running buffer (Invitrogen) and then soaked in MOPS SDS running buffer twice for 30 min before electrotransfer. FLAG and GFP were detected with antibodies from Sigma and Roche respectively.

### RNase E activity

Expression of full‐length RNase E and cRNase E with C‐terminal His tags, purification under denaturing conditions on Ni‐NTA affinity columns and assay of endo‐ribonuclease activity using the radioactively labelled 9Sa rRNA substrate were as described (Khemici *et al.*, 2008). Rne(1‐598) and Rne(1‐598)*∆*MTS with C‐terminal His tags were purified under native conditions. Expression was induced with IPTG in BL21(DE3) pLysS as described (Khemici *et al.*, 2008). Cell extracts were prepared from 200 ml cultures with minor modification of the small‐scale protocol as described (Carpousis *et al.*, 2001).

IMAC purification was based on a previously described protocol with minor modifications (Worrall *et al.*, 2008). cOmplete EDTA‐free protease inhibitors (Roche) were used throughout. Lysates were clarified by centrifugation in a Beckman 70 Ti rotor at 4°C: 18,000 rpm, 60 min and then 45,000 rpm, 60 min. A 1 ml HisTrap FF column was equilibrated with buffer A (10 mM Tris HCl, pH 7.5, 500 mM NaCl, 5% glycerol, 0.2% Genapol X‐080, 10 mM MgSO_4,_ 1 mM EDTA, 1 mM TCEP, 1x protease inhibitors) containing 12 mM imidazole (pH 7.5). After loading, the column was washed with buffer A containing 1 M urea and then buffer A containing 60 mM imidazole. The protein was eluted with a 60–600 mM imidazole gradient. The peak fractions were pooled, dialyzed against buffer A with 50% glycerol and stored at −80°C. Protein concentrations were determined using an extinction coefficient of 28,880 (0.1%, 280 nm, https://web.expasy.org/protparam/).

RNase E cleavage of the 9Sa‐Cy5 substrate was measured using modifications of a previously described protocol (Khemici *et al.*, 2008). Enzyme buffer (EB) contained Genapol X‐80 instead of Triton X‐100 and BSA was omitted. Yeast RNA was omitted from substrate buffer (SB), which was supplemented with 1.25 U/µl RiboLock RNase Inhibitor (Thermo Scientific). The final concentration of MgCl_2_ was 8 mM. The 9Sa‐Cy5 substrate, which was synthesized by Dharmacon, was suspended in water to a concentration of 100 µM and stored at −20°C. Prior to use, an aliquot of the 9Sa‐Cy5 substrate was diluted in water (typically 20 µM) and heated in a thermocycler to 65°C for 5 min, cooled to 4°C for 1 min, and then diluted into SB. Final concentration of substrate ranged from 0.5 to 15 µM. Reactions were performed with 10 or 20 nM enzyme to limit the extent of cleavage (≤20%). After incubation at 37°C for 10 minutes, the reactions were chilled on ice, diluted with an equal volume of 2x loading buffer (6% Ficoll, 10 mM Tris‐HCl, pH 7.5, 20 mM EDTA, 0.4% SDS, 0.01% BPB), and then incubated at 50°C for 10 minutes. Half of the reaction (10 µl) was loaded on 20% TBE (15‐well) gels (InVitrogen). Electrophoresis in TBE was at 160 V until the BPB was 1 cm from the bottom. Gels were scanned on a Typhoon biomolecular imager (Amersham). Cy5 fluorescence was quantified with ImageQuant software. The proportion of product to total was used to calculate the rate of cleavage.

### CsrB/C stability

*E. coli* strains were grown either in LB or M9‐glucose medium at 37°C to an OD_600_ of 0.6 and then rifampicin was added to a final concentration of 500 µg ml^−1^. Aliquots were withdrawn at times ranging from 1 to 32 min, mixed with 0.2 volume of stop solution (5% phenol, 95% ethanol v/v), and frozen in liquid nitrogen. Total RNA was extracted from pelleted cells using TRIzol® reagent. CsrB and CsrC were analysed by northern blotting using riboprobes as described (Suzuki *et al.*, 2006).

### Expression of *glgC* and glycogen levels

Wild type and *rne∆MTS* strains, transformed with pLH40, were grown on LB at 37°C to an OD_600_ of 0.6 or 2. Cells were pelleted by centrifugation, suspended in SDS‐PAGE loading buffer and lysed by heating. GFP was detected by western blotting using anti‐GFP antibody (Roche). To measure glycogen levels, single colonies were streaked or patched onto an LB agar plate supplemented with 2% glucose and incubated overnight at 37°C. Glycogen was detected by staining with iodine vapor for 2 min at 37°C.

### Data analysis

Half‐life data were graphed in Excel using exponential fitting and semi‐log plotting. Half‐lives were calculated as the average and standard deviation of at least three replicates. In Fig. 5D, the data was fitted to the Michaelis–Menten equation using the Solver function of Excel. K_m_ and k_cat_ were calculated as the average and standard deviation of three replicates. In Figs 1, 2 and 7, associated *p*‐values were calculated using Welch’s t‐test.

## Author contributions

LP, MB and AJC initiated the project. LH, LP, MB, MCB, LG and AJC designed experiments and analysed data. LH, LP, MB, QMO, IC and AJC performed the experimental work. LH, LP and AJC wrote the manuscript with feedback from the other authors.
