# Supplementary material for: Detachment of the RNA degradosome from the inner membrane of Escherichia coli results in a global slowdown of mRNA degradation, proteolysis of RNase E and increased turnover of ribosome‐free transcripts
Source: Mol Microbiol. 2019 Apr 6;111(6):1715–31. doi: 10.1111/mmi.14248 (PMC6850036; doi:10.1111/mmi.14248)
Supplement: Supplementary file 1 [file MMI-111-1715-s001.pdf]

Supporting information

Detachment of the RNA degradosome from the inner membrane of *Escherichia coli* results in a global slowdown of mRNA degradation, proteolysis of RNase E and increased turnover of ribosome-free transcripts.

Lydia Hadjeras[1*], Leonora Poljak[1*], Marie Bouvier[1], Quentin Morin-Ogier[1], Isabelle Canal[1], Muriel Cocaign-Bousquet[2], Laurence Girbal[2†], and Agamemnon J. Carpousis[1†]

[1]Laboratoire de Microbiologie et de Génétique Moléculaires, Centre de Biologie Intégrative (CBI), Université de Toulouse, CNRS, UPS, France

[2] LISBP, Université de Toulouse, CNRS, INRA, INSA, Toulouse, France

Running title: **Cell biology of RNA degradation in *E. coli***

*These authors contributed equally to this work.

[†]Corresponding author: girbal@insa-toulouse.fr, carpousi@ibcg.biotoul.fr

Table S1. Strains and plasmids.

**Strain**	**Genotype**	**Reference**
MG1655	F$^-$, λ$^-$, *rph*$^-$	(Jensen, 1993)
NCM3416	F$^-$, λ$^-$, *zib-207*::Tn*10*	(Soupene *et al.*, 2003)
BL21(DE3)pLysS	F$^-$, *omp*T, *hsd*S$_B$ (r$_B^-$, m$_B^-$), *dcm*, *gal*, λ(DE3), pLysS, Cm^r	(Studier & Moffatt, 1986)
Kti658	MG1655, *rne*Δ*MTS-frt*	this work
Kti578	MG1655, *csrA*51	(Esquerre *et al.*, 2016)
Kti754	MG1655, *csrA*51 *rne*Δ*MTS-fr-cat-frt*	this work
Kti684	MG1655, Δ*csrD*	(Esquerre *et al.*, 2016)
MBS106	NCM3416, *rne-frt*	this work
MBS157	NCM3416, *rne*Δ*MTS-frt*	this work
TM338	W3110, *rne-flag-frt-cat-frt*	(Morita *et al.*, 2005)
LHS393	NCM3416, *rne-flag-frt*	this work
LHS395	NCM3416, *rne*Δ*MTS-flag-frt*	this work
LHS420	NCM3416, *rne-flag-frt*, *rhlB-gfp-frt*	this work
LHS422	NCM3416, *rne*Δ*MTS-flag-frt*, *rhlB-gfp-frt*	this work
LHS554	MG1655, *rne-flag-frt*	this work
LHS556	MG1655, *rne*Δ*MTS-flag-frt*	this work
ENS133	BL21(DE3), *lacZ*::Tn*10*, *malP*pΔ*534*::P$_{lac}$*lacZ-Arg5*	(Lopez *et al.*, 1994)
ENS134	BL21(DE3), *lacZ*::Tn*10*, *malP*pΔ*534*::P$_{T7}$*lacZ-Arg5*	(Lopez *et al.*, 1994)
MGM133.1	ENS133, *rne-flag-frt*	this work
MGM133.2	ENS133, *rne*Δ*MTS-flag-frt*	this work
MGM134.1	ENS134, *rne-flag-frt*	this work
MGM134.2	ENS134, *rne*Δ*MTS-flag-frt*	this work
MGM134.11	ENS134, *rne-flag-frt*, *rhlB*$^-$	this work
MGM134.21	ENS134, *rne*Δ*MTS-flag-frt*, *rhlB*$^-$	this work
MGM134.12	ENS134, *rne-flag-frt*, *pnp*$^-$	this work
MGM134.22	ENS134, *rne*Δ*MTS-flag-frt*, *pnp*$^-$	this work
LHS570	NCM3416, *rne-flag-frt* Δ*glgC-frt*	this work
LHS572	NCM3416, *rne*Δ*MTS-flag-frt* Δ*glgC-frt*	this work
**Plasmid**	**Features**	**Reference**
pEZ201	pSC101 origin, ampicillin resistance	(Jain & Belasco, 1995)
pEZ206	pSC101 origin, ampicillin resistance	(Jain & Belasco, 1995)
pXG10	pSC101 origin, *gfp* coding sequence, chloramphenicol resistance	(Urban & Vogel, 2007)
pLH40	pXG10, *glgC* expression signals fused to *gfp* coding sequence	this work
pSAB11	pSC101 origin, *rne* expression signals, spectinomycin resistance	(Ait-Bara & Carpousis, 2010)
pLH43	pSAB11, *rne* transcription signals fused to *lacZ* coding sequence	this work
pET21b-*rne*	*rne* coding sequence with C-terminal His-tag	(Khemici *et al.*, 2008)
pLP56-2	pET-*rne*(1-598)-his6	this work
pLP57-2	pET-*rne*(1-598)-ΔMTS-his6	this work

Table S2. DNA probes for RNA blots.

Name	Sequence (5' – 3')
tRNA$_{arg5}$	TGGAGTCCCCTGCAG
23S rRNA	CAGCGTGCCTTCTCCCGAAG
rplU	GAACGGTCTGACCTTCGCTTACTC
rpmB	AGTAACTTGGCAGACTCGGGACAT
rpsT1	CTTGCGTTGTGCTTACGAGCCTTT
rpsT2	GCTTTGTCGCCAGCTTCGATAGCT
trxA1	CCGCTTTGAGTACATCCGTGTCAA
trxA2	GCCCTGATATTCGTCAGCGATTTC
rpsO1	TGCGTCACGACCAAACTCAGAAAC
rpsO2	AGCAGTGCTACCTGAACTTCGGTA

Table S3. Excel file of slot blot data.

Table S4. Excel file of GO data.

Table S5. Excel file of 246 ORFs.

Table S6. Autoregulation of *rne* mRNA stability.

Strain	Genotype	Medium	Temp.	pEZ201	Δ*MTS*/*rne*[+]	pEZ206	Δ*MTS*/*rne*[+]	pLH43	Δ*MTS*/*rne*[+]
				units[a]	ratio	units	ratio	units	ratio
MBS106	*rne frt*[b]	M9-glucose	37 °C	1,110±10		1,850±30		56,080±1090	
MBS157	*rne*Δ*MTS frt*	M9-glucose	37 °C	2,230±60	2.01	1,870±90	0.99	57,610±1390	0.98
MG1655	*rne*	M9-glucose	37 °C	1,350±70		1,930±30		66,780±1640	
Kti658	*rne*Δ*MTS frt*	M9-glucose	37 °C	2,220±30	1.64	1,770±30	1.09	65,220±1000	1.02
MBS106	*rne frt*	MOPS-glycerol-CAA[c]	30 °C	1,820±60		3,480±30		55,670±800	
MBS157	*rne*Δ*MTS frt*	MOPS-glycerol-CAA	30 °C	2,450±40	1.35	2,620±40	1.33	43,920±2100	1.28
MG1655	*rne*	MOPS-glycerol-CAA	30 °C	2,640±30		3,760±70		64,630±2940	
Kti658	*rne*Δ*MTS frt*	MOPS-glycerol-CAA	30 °C	3,440±70	1.30	2,940±150	1.28	49,680±2630	1.30
MBS106	*rne frt*	LB	37 °C	2,740±100		3,260±30		27,940±530	
MBS157	*rne*Δ*MTS frt*	LB	37 °C	4,120±50	1.50	2,790±10	1.17	26,810±950	1.04
MG1655	*rne*	LB	37 °C	2,420±120		3,210±120		29,390±1400	
Kti658	*rne*Δ*MTS frt*	LB	37 °C	4,250±90	1.76	2,950±40	1.09	30,840±810	0.95

[a]Miller units (Zhang & Bremer, 1995): mean and standard deviation of three replicates. [b]*Frt* (FLP recognition target) indicates 'scar' sequence located downstream of the *rne* gene, which is formed upon removal the drug resistant cassette by FLP recombinase (Datsenko & Wanner, 2000). [c]Casamino acids.

Table S7. RNase E levels.

Strain	Genotype	Medium	Temp	Level[a]
LHS393	NCM3416, *rne-flag-frt*[b]	MOPS-glycerol-CAA[c]	30 °C	0.71±0.08
LHS395	NCM3416, *rneΔMTS-flag-frt*	MOPS-glycerol-CAA	30 °C	0.63±0.10
LHS554	MG1655, *rne-flag-frt*	MOPS-glycerol-CAA	30 °C	1.10±0.17
LHS556	MG1655, *rneΔMTS-flag-frt*	MOPS-glycerol-CAA	30 °C	1.27±0.09
LHS393	NCM3416, *rne-flag-frt*	M9-glucose	37 °C	0.99±0.08
LHS395	NCM3416, *rneΔMTS-flag-frt*	M9-glucose	37 °C	1.03±0.04
LHS554	MG1655, *rne-flag-frt*	M9-glucose	37 °C	1.29±0.22
LHS556	MG1655, *rneΔMTS-flag-frt*	M9-glucose	37 °C	1.30±0.07
LHS393	NCM3416, *rne-flag-frt*	LB	37 °C	0.80±0.08
LHS395	NCM3416, *rneΔMTS-flag-frt*	LB	37 °C	0.70±0.05
LHS554	MG1655, *rne-flag-frt*	LB	37 °C	0.75±0.16
LHS556	MG1655, *rneΔMTS-flag-frt*	LB	37 °C	0.71±0.07

[a]Mean and standard deviation of three replicates. [b]*Frt* (FLP recognition target) indicates ‘scar’ sequence located downstream of the *rne* gene, which is formed upon removal the drug resistant cassette by FLP recombinase (Datsenko & Wanner, 2000). [c]Casamino acids.

Table S8. Stability of the T7-*lacZ* mRNA.

**Strain**[a]	**Genotype**	**β-galactosidase**	**tRNA$_{arg5}$**
P$_{lac}$			
MGM133.1	*rne*$^{+}$	5410±460	nd
MGM133.2	*rne*ΔMTS	5650±205	nd
P$_{T7}$			
MGM134.1	*rne*$^{+}$	3120±50	510±90
MGM134.2	*rne*ΔMTS	430±80	660±120
MGM134.11	*rne*$^{+}$ *rhlB*$^{-}$	9320±1070	600±90
MGM134.12	*rne*$^{+}$ *pnp*$^{-}$	6720±1450	620±160
MGM134.21	*rne*ΔMTS *rhlB*$^{-}$	2170±430	740±140
MGM134.22	*rne*ΔMTS *pnp*$^{-}$	1620±880	620±150

[a]Isogenic set of strains in ENS133 & ENS134 background (Lopez *et al.*, 1994). Miller units (Zhang & Bremer, 1995): mean and standard deviation of at least three replicates. Level of tRNA$_{arg5}$ expressed as arbitrary units normalized to 16S rRNA. Not determined (nd).

References

Ait-Bara, S., and Carpousis, A.J. (2010) Characterization of the RNA degradosome of Pseudoalteromonas haloplanktis: conservation of the RNase E-RhlB interaction in the gammaproteobacteria. *J Bacteriol* **192**: 5413-5423.

Datsenko, K.A., and Wanner, B.L. (2000) One-step inactivation of chromosomal genes in Escherichia coli K-12 using PCR products. *Proc Natl Acad Sci U S A* **97**: 6640-6645.

Deana, A., Celesnik, H., and Belasco, J.G. (2008) The bacterial enzyme RppH triggers messenger RNA degradation by 5' pyrophosphate removal. *Nature* **451**: 355-358.

Esquerre, T., Bouvier, M., Turlan, C., Carpousis, A.J., Girbal, L., and Cocaign-Bousquet, M. (2016) The Csr system regulates genome-wide mRNA stability and transcription and thus gene expression in Escherichia coli. *Sci Rep* **6**: 25057.

Jain, C., and Belasco, J.G. (1995) RNase E autoregulates its synthesis by controlling the degradation rate of its own mRNA in Escherichia coli: unusual sensitivity of the rne transcript to RNase E activity. *Genes Dev* **9**: 84-96.

Jensen, K.F. (1993) The Escherichia coli K-12 "wild types" W3110 and MG1655 have an rph frameshift mutation that leads to pyrimidine starvation due to low pyrE expression levels. *J Bacteriol* **175**: 3401-3407.

Khemici, V., Poljak, L., Luisi, B.F., and Carpousis, A.J. (2008) The RNase E of Escherichia coli is a membrane-binding protein. *Mol Microbiol* **70**: 799-813.

Lopez, P.J., Iost, I., and Dreyfus, M. (1994) The use of a tRNA as a transcriptional reporter: the T7 late promoter is extremely efficient in Escherichia coli but its transcripts are poorly expressed. *Nucleic Acids Res* **22**: 2434.

Moffitt, J.R., Pandey, S., Boettiger, A.N., Wang, S., and Zhuang, X. (2016) Spatial organization shapes the turnover of a bacterial transcriptome. *eLife* **5**.

Morita, T., Maki, K., and Aiba, H. (2005) RNase E-based ribonucleoprotein complexes: mechanical basis of mRNA destabilization mediated by bacterial noncoding RNAs. *Genes Dev* **19**: 2176-2186.

Soupene, E., van Heeswijk, W.C., Plumbridge, J., Stewart, V., Bertenthal, D., Lee, H., Prasad, G., Paliy, O., Charernnoppakul, P., and Kustu, S. (2003) Physiological studies of Escherichia coli strain MG1655: growth defects and apparent cross-regulation of gene expression. *J Bacteriol* **185**: 5611-5626.

Studier, F.W., and Moffatt, B.A. (1986) Use of bacteriophage T7 RNA polymerase to direct selective high-level expression of cloned genes. *J Mol Biol* **189**: 113-130.

Urban, J.H., and Vogel, J. (2007) Translational control and target recognition by Escherichia coli small RNAs in vivo. *Nucleic Acids Res* **35**: 1018-1037.

Zhang, X., and Bremer, H. (1995) Control of the Escherichia coli rrnB P1 promoter strength by ppGpp. *J Biol Chem* **270**: 11181-11189.

FIGURES LEGENDS

Fig. S1. Gene Ontology.

Functional analysis of the ORFs selected in the Volcano plot showed that 13 Gene Ontology (GO) terms were enriched (p-value<0.05). The enriched GO terms are ranked by p-value. Segment size is proportional to the number of genes associated with the corresponding GO term.

Fig. S2. Differential stability of mRNA.

A. 442 ORFs encoding Inner Membrane Proteins (IMPs) (Moffitt *et al.*, 2016).

B. 275 ORFs corresponding to mRNAs that are destabilized by RppH (Deana *et al.*, 2008).

C. 290 ORFs encoding proteins that are targeted to the inner cytoplasmic membrane by the Signal Recognition Particle (SRP) (Moffitt *et al.*, 2016).

D. 163 ORFs encoding proteins that are targeted to the inner cytoplasmic membrane by the SecB pathway (Moffitt *et al.*, 2016).

Fig. S3. Quantitative Western blotting of RNase E levels.

Total protein extracts were prepared from isogenic pairs of strains encoding wild type RNase E or cRNase E with a C-terminal FLAG tag. The strains were grown under three different conditions. S1, S2, S3 and S4 are internal standards used to normalize levels between blots representing three replicates.

Fig. S4. CsrB and CsrC stability.

Northern blots of the stability of CsrB and CsrC. Strains were grown on M9-glucose at 37 °C to an OD_{600} = 0.4. Total RNA was extracted prior to or at various times after the addition of rifampicin. RNA levels were quantified by Northern blotting. Half-lives of CsrB and CsrC are shown as the mean and standard deviation of three replicates.

Fig. S5. Northern blots of $tRNA_{arg5}$.

Northern blots of RNA prepared from the same cultures used to measure β-galactosidase levels in Fig. 7C. Equal amounts of total RNA were separated by gel electrophoresis. In the upper panel, the blots were probed with a ^{32}P-labelled oligonucleotide specific to $tRNA_{arg5}$. In the lower panel, the blot were stripped and the hybridized with a probe specific to 16S rRNA.

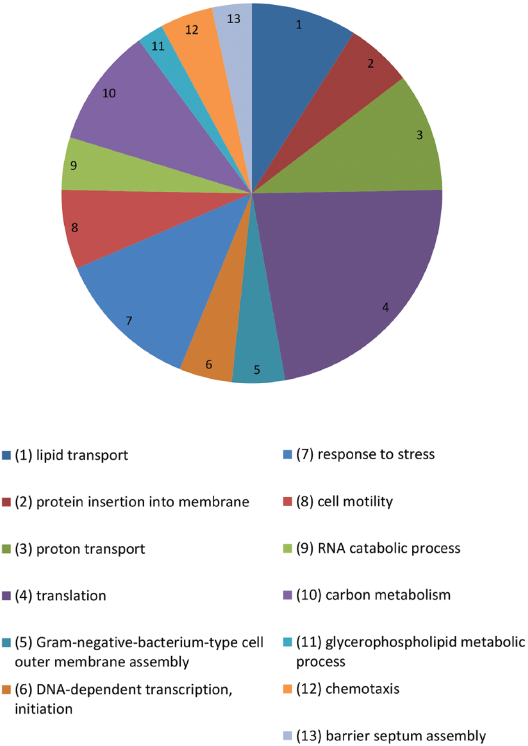
1
2
3
4
5
6
7
8
9
10
11
12
13
(1) lipid transport
(2) protein insertion into membrane
(3) proton transport
(4) translation
(5) Gram-negative-bacterium-type cell outer membrane assembly
(6) DNA-dependent transcription, initiation
(7) response to stress
(8) cell motility
(9) RNA catabolic process
(10) carbon metabolism
(11) glycerophospholipid metabolic process
(12) chemotaxis
(13) barrier septum assembly

Figure S1

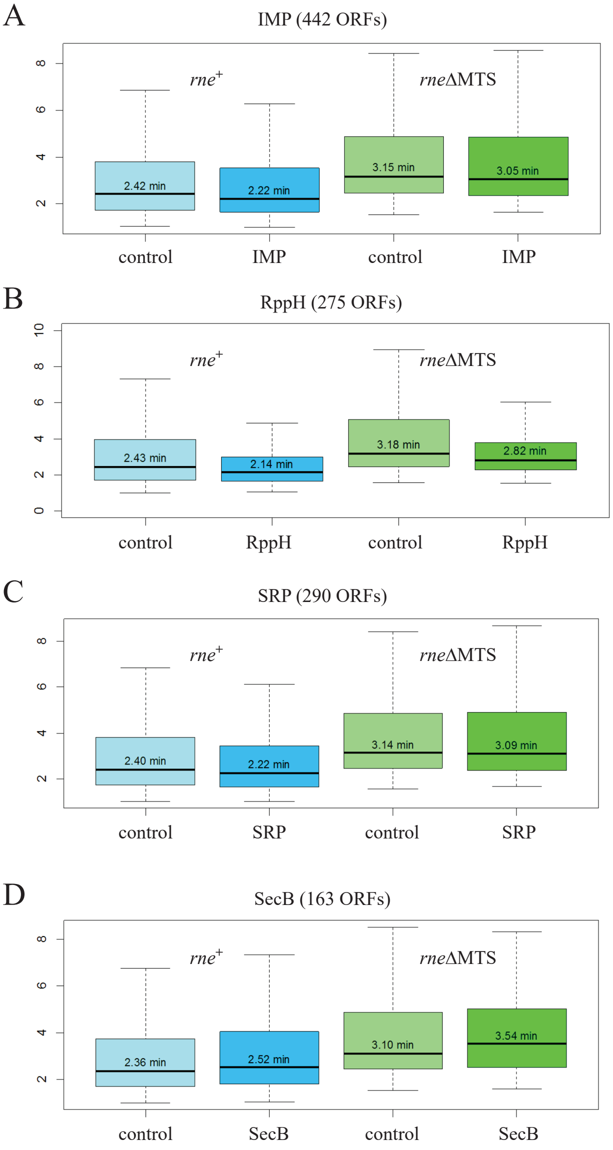
A
IMP (442 ORFs)
rne+
rneΔMTS
2.42 min
2.22 min
3.15 min
3.05 min
control
IMP
control
IMP
B
RppH (275 ORFs)
rne+
rneΔMTS
2.43 min
2.14 min
3.18 min
2.82 min
control
RppH
control
RppH
C
SRP (290 ORFs)
rne+
rneΔMTS
2.40 min
2.22 min
3.14 min
3.09 min
control
SRP
control
SRP
D
SecB (163 ORFs)
rne+
rneΔMTS
2.36 min
2.52 min
3.10 min
3.54 min
control
SecB
control
SecB

Figure S2

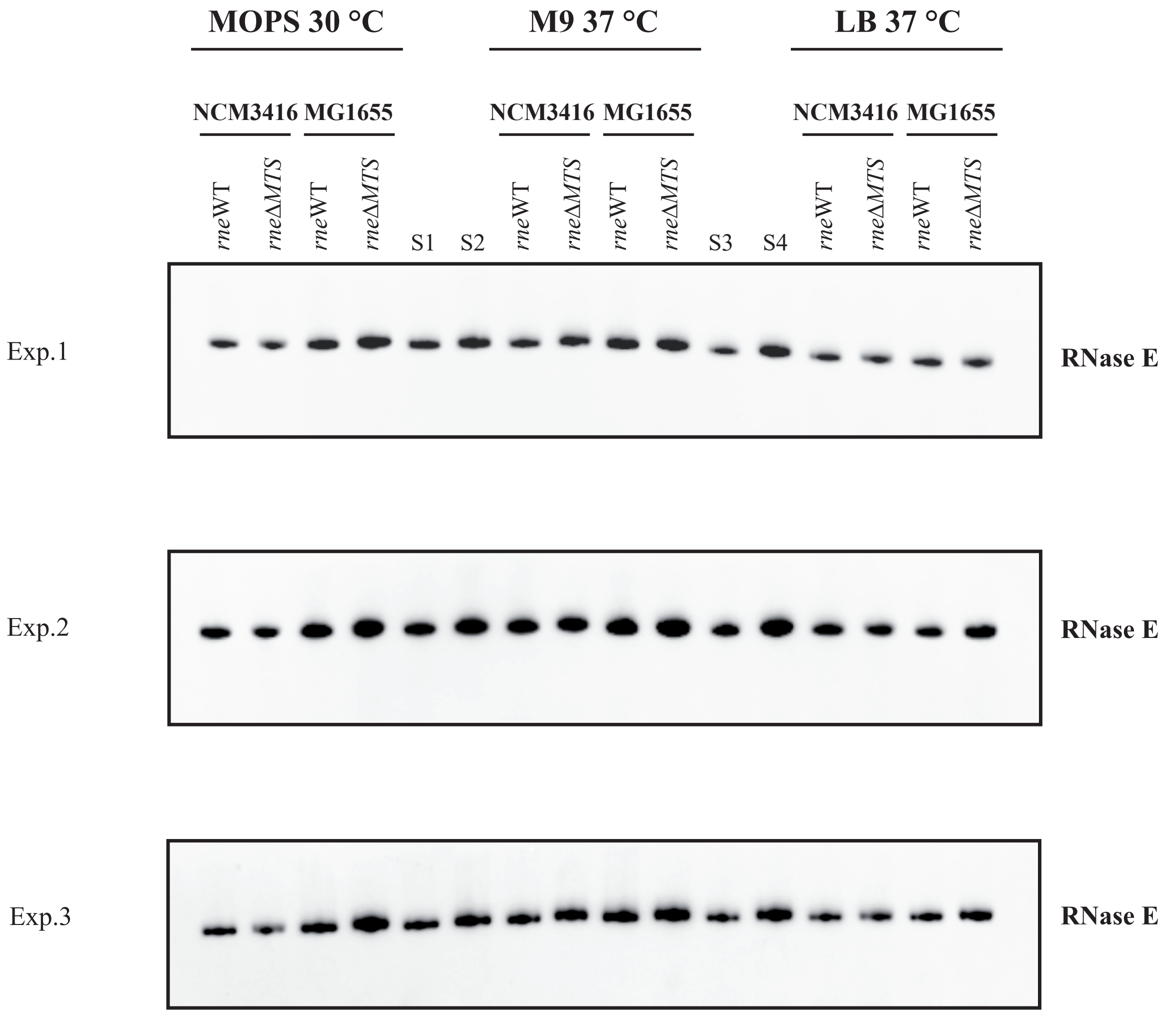
MOPS 30 °C
M9 37 °C
LB 37 °C
NCM3416
MG1655
NCM3416
MG1655
NCM3416
MG1655
rneWT
rneΔMTS
rneWT
rneΔMTS
S1
S2
rneWT
rneΔMTS
rneWT
rneΔMTS
S3
S4
rneWT
rneΔMTS
rneWT
rneΔMTS
Exp.1
RNase E
Exp.2
RNase E
Exp.3
RNase E

Figure S3

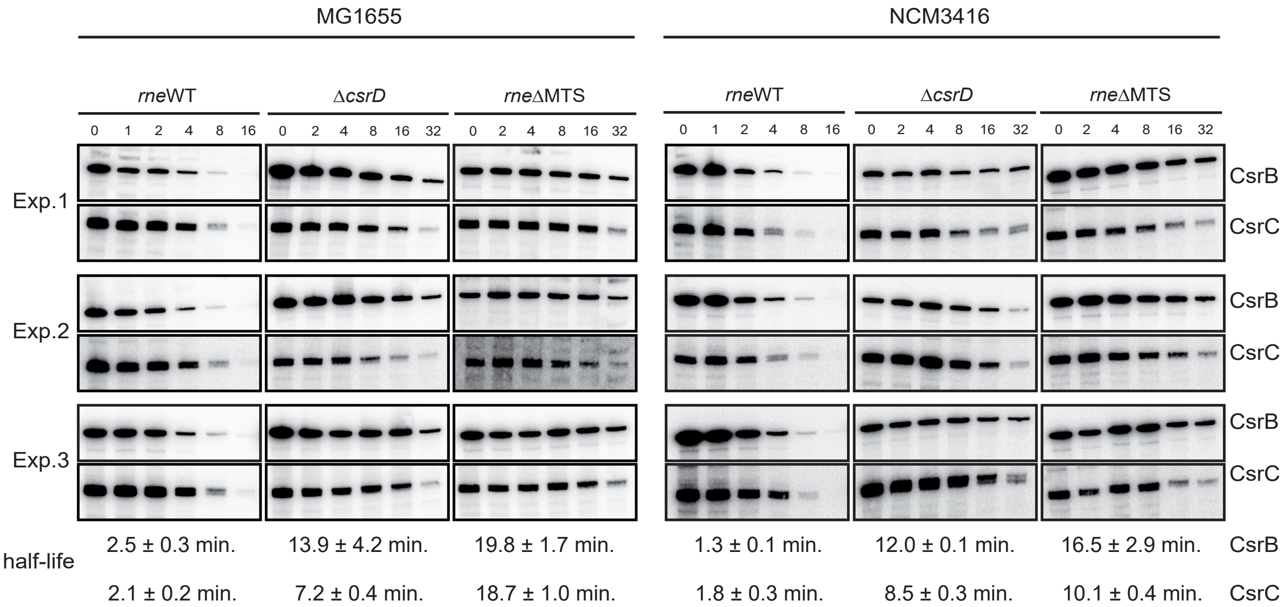
MG1655
NCM3416
rneWT
ΔcsrD
rneΔMTS
rneWT
ΔcsrD
rneΔMTS
0 1 2 4 8 16
0 2 4 8 16 32
0 2 4 8 16 32
0 1 2 4 8 16
0 2 4 8 16 32
0 2 4 8 16 32
Exp.1
Exp.2
Exp.3
CsrB
CsrC
CsrB
CsrC
CsrB
CsrC
half-life
2.5 ± 0.3 min.
13.9 ± 4.2 min.
19.8 ± 1.7 min.
1.3 ± 0.1 min.
12.0 ± 0.1 min.
16.5 ± 2.9 min.
CsrB
2.1 ± 0.2 min.
7.2 ± 0.4 min.
18.7 ± 1.0 min.
1.8 ± 0.3 min.
8.5 ± 0.3 min.
10.1 ± 0.4 min.
CsrC

Figure S4

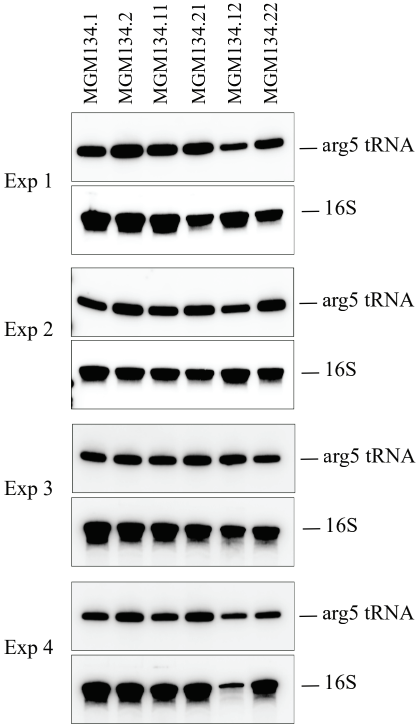
MGM134.1
MGM134.2
MGM134.11
MGM134.21
MGM134.12
MGM134.22
arg5 tRNA
Exp 1
16S
arg5 tRNA
Exp 2
16S
arg5 tRNA
Exp 3
16S
arg5 tRNA
Exp 4
16S

Figure S5
